# Refining Bioequivalence Assessment of Topical Drug Products for Local Action: A Comparative Analysis of Tape Stripping Methodologies

**DOI:** 10.3390/pharmaceutics18020194

**Published:** 2026-02-02

**Authors:** Seeprarani Rath, Isadore Kanfer

**Affiliations:** 1 Center for Dermal Research, Rutgers-The State University of New Jersey, 145 Bevier Road, Piscataway, NJ 08854, USA; seeprapr@gmail.com; 2 Biopharmaceutics Research Institute, Rhodes University, Grahamstown 6139, South Africa; 3 Leslie Dan Faculty of Pharmacy, University of Toronto, Toronto, ON M5S 3M2, Canada

**Keywords:** tape stripping, dermatopharmacokinetics (DPK), topical bioequivalence, locally acting topical drug products, stratum corneum drug uptake, uptake-only (single time point) study design, uptake-clearance (two time point) study design, transepidermal water loss (TEWL), skin thickness normalization

## Abstract

Tape stripping (TS) is a minimally invasive technique that enables in vivo assessment of drug uptake in the skin of human subjects. Whilst appropriate for evaluating the bioequivalence (BE) of topical formulations, methodological variations persist, especially regarding the inclusion of clearance time measurements. This manuscript compares the TS protocols described in several publications, including the European Medicines Agency (EMA) and Japanese guidance, containing information relating to the evaluation of utility, practicality, and scientific validity. Evidence suggests that for drugs acting locally in the skin, clearance measurements that require time-consuming practical manipulation and subsequent complex data processing offer limited value, whereas single time point uptake studies, which are more convenient and expedient, may suffice for regulatory BE assessments. This discussion offers practical guidance for BE assessment of topical dermatological products and a more expedient approach using a single time point uptake study protocol, obviating the need for clearance time assessments.

## 1. Introduction

The stratum corneum (SC), the skin’s outermost layer, provides the primary barrier to percutaneous drug penetration, transport, and distribution. This specialized nature of the SC presents a significant hurdle for topical drug delivery, making cutaneous drug permeation a slow and complex process where penetration through this layer often dictates the rate at which a drug can exert its therapeutic effects. Consequently, the development and evaluation of topical drug products necessitate robust methodologies to assess in vivo drug uptake into the skin [1]. Assessing drug bioavailability (BA) and bioequivalence (BE) in skin is essential for topical dermatological product development. Tape stripping (TS) has become a cornerstone methodology due to its minimal invasiveness and ability to quantify drug uptake and penetration in situ [1,2], thereby offering a considerable advantage over more invasive techniques, providing a more comfortable experience for patients while still yielding reliable data. The introduction of the TS method marks a significant advancement in experimental tools for skin research [1,2]. This characteristic is crucial in expanding the scope of skin research, allowing for repeated sampling from the same skin site [3]. The versatility of the TS methodology makes it applicable to a wide spectrum of dermatological conditions, especially those where the primary barrier, the SC, is either the site of the disease or the rate-limiting step for drug delivery in psoriasis, atopic dermatitis, acne, and superficial fungal infections. It is particularly relevant to topical therapies that act primarily within or near the stratum corneum and where the quantitative assessment of local drug uptake is clinically meaningful [4,5,6,7,8,9]. However, methodological disparities, particularly regarding the assessment of clearance time, have generated debate about the optimal TS design for BE assessment of topical products not intended to be absorbed into the systemic circulation [10,11]. This work introduces a decision-tree framework to guide design selection between single-time-point and dual-time-point TS approaches based on the site of drug action and regulatory context.

### Limitations of Applying Systemic Pharmacokinetic Metrics to Topical Drug Products

Concerns have long been raised regarding the appropriateness of directly applying conventional pharmacokinetic parameters, such as area under the concentration–time curve (AUC) and maximum concentration (C_max_), to the assessment of BE for topical drug products. These parameters are rooted in the principles of oral pharmacokinetics and systemic drug disposition and, therefore, may not adequately reflect the processes governing drug delivery following topical administration.

For orally administered dosage forms, the plasma concentration–time profile is determined by a sequence of well-defined systemic processes, including absorption from the gastrointestinal tract, distribution into tissues, metabolism, and elimination. In contrast, following topical application, drug disposition is primarily governed by penetration into and within the skin, particularly the SC, and is controlled by local physicochemical and biological processes such as partitioning, diffusion, binding to keratin, and retention within cutaneous layers. Consequently, the concentration at the topical site of action is largely dictated by SC penetration kinetics rather than systemic exposure.

In addition, the temporal characteristics of drug exposure differ fundamentally between oral and topical routes. In a biostudy, after topical application, penetration into the SC begins immediately, but the formulation is typically removed after a predefined exposure period, thereby abruptly terminating further drug input. By contrast, for immediate-release oral products, absorption continues until the dosage form is depleted, after which elimination processes predominate. These differences result in pharmacokinetic profiles for topical products that are inherently distinct from those observed following systemic administration.

Taken together, these considerations underscore that oral and topical dosage forms exhibit fundamentally different kinetic behaviors, and that uncritical adoption of systemic PK metrics for topical BE assessment may be scientifically inappropriate. This conceptual distinction forms the basis for ongoing efforts to develop dermatopharmacokinetic (DPK) approaches and fit-for-purpose metrics that better reflect local drug delivery and action within the skin [12,13].

## 2. Principles and Applications of Tape Stripping

### 2.1. Fundamental Technique

Tape stripping involves the sequential removal of microscopic layers (0.5–1 µm) of the SC using adhesive tape applied under standardized pressure [1,14,15]. This allows measurement of drug concentration profiles across SC depth [1,14,15]. The technique supports regulatory BE evaluations, dermatological drug development, exposure assessment, and skin physiology studies [6,14,16,17].

### 2.2. Bioequivalence via TS

Bioequivalence (BE) via TS hinges on comparing drug uptake into the skin between test and reference formulations [1,14,15]. Pharmacokinetic (PK) parameters such as area under the curve (AUC) and drug amount measurable in the SC samples are central to these comparisons [14,15,16].

## 3. Methodological Review

### 3.1. Rigorous BE Protocols Developed at the Biopharmaceutics Research Institute (BRI)

The Biopharmaceutics Research Institute (BRI), led by Professor Kanfer and colleagues, was pivotal in establishing TS as a structured, statistically powered tool for BE. The BRI is a Contract Research Organization that conducts BA, BE, and PK studies in line with international standards. Following good clinical practice, it provides clinical, statistical, and reporting services to support the development and approval of new and generic medicines [18]. Kanfer and colleagues at the BRI [18] focused on using single time point TS methods to assess BE of topical products [4,5,11,12,19,20,21,22,23,24,25,26,27].

**Figure 1:**
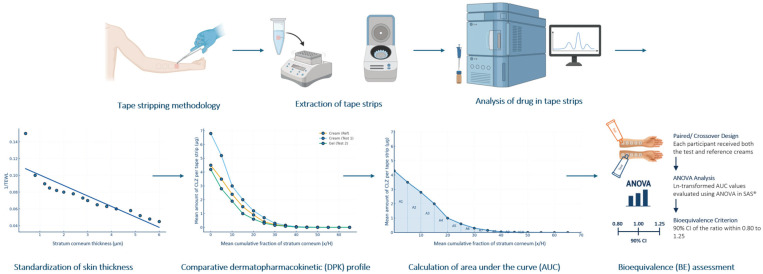
Overview of the TS methodology at the BRI.

The methodology employed (Figure 1) involves a series of well-defined procedural steps. The study designs include an initial pilot study to establish the optimal application time for the topical product through dose duration studies, ensuring the sensitivity of the TS method in detecting significant differences between formulations. Typically, the application site is the volar aspect of the forearm, a well-established and consistent area for dermatological testing. A weighed dose (e.g., ~15 mg/cm^2^) of the topical formulation is applied to a demarcated square area on the forearm (e.g., 2 × 2 cm^2^) and left in contact with the skin for a specific duration, which varies depending on the study. Following the designated contact time, any excess formulation remaining on the application sites is carefully removed using dry cotton buds or by swabbing to ensure that only the drug that had penetrated into the skin is measured. Fifteen to twenty tape strips are applied with uniform pressure and removed sequentially with directional rotation to minimize bias [5]. The subsequent data analysis involves excluding the first tape strip from the primary analysis, as it is considered likely to contain residual unabsorbed drug product that has not been effectively removed from the skin surface. Each of the remaining tape strips is extracted, and the amount of drug in each tape strip is determined using high-performance liquid chromatography (HPLC) [4,5,11,23]. To account for intersubject variability in SC thickness, it is necessary to normalize the differences in SC thickness between subjects. This approach expresses drug concentration profiles relative to the fraction of the SC removed rather than the absolute depth, which can differ markedly among individuals due to variations in skin hydration, anatomical site, and physiological factors. Representing depth as a fraction of total SC thickness enables direct comparison of profiles across subjects, providing a consistent basis for evaluating drug penetration behavior. Normalized SC thickness is estimated from the cumulative mass removed during TS and used as the normalized depth coordinate for plotting drug concentration profiles. The cumulative fraction of the SC removed, expressed as x/H, is commonly used to normalize drug distribution profiles obtained from TS studies. In this approach, x represents the cumulative depth of the SC removed, and H denotes the total SC thickness. The total SC thickness (H) can be estimated from transepidermal water loss (TEWL) measurements, following the relationship originally described by Maibach and colleagues [28]. TEWL measurements are used to normalize SC thickness between the healthy human study participants [29]. The measurements are taken using a vapometer after successive tape strips have been removed, and the data are fitted to the equation:

(1) $$\frac{1}{{TEWL}_{X}}=\;\frac{H}{K\cdot \Delta C\cdot D}-\;\frac{x}{K\cdot \Delta C\cdot D}\;$$

where *TEWL_X_* is the transepidermal water flux after removal of a cumulative thickness *x* of SC, *K* is the partition coefficient of water between the SC and viable tissue, *D* is the apparent diffusivity of water, and Δ*C* is the water concentration gradient across the membrane. Since *K*, *D*, and Δ*C* are constants, this relationship can be simplified to a linear form:

(2) $$\frac{1}{{TEWL}_{X}}=\;H-x$$

Assuming the SC material adhering to each tape strip is uniform and has a density of approximately 1 g/cm^3^, the cumulative depth x can be calculated from the cumulative SC mass removed. The total SC thickness (*H*) is then obtained from the x-intercept of the linear regression of 1/*TEWL_X_* versus *x.* Once *H* is determined, the normalized cumulative depth *x/H* is calculated for each tape strip. This parameter represents the relative position within the SC, ranging from 0 at the surface to 1 at the SC and viable epidermis interface. Drug concentration or amount per strip is then plotted as a function of *x/H* to generate drug distribution profiles across the SC. The area under the curve (AUC) of this plot is calculated using the trapezoidal rule to quantify total drug uptake within the SC. The resulting AUC values serve as comparative metrics in DPK evaluations. Bioequivalence between test and reference products is then assessed statistically by comparing these AUC values (both TEWL-corrected and uncorrected) using the 90% confidence interval for the test/reference ratio, consistent with FDA guidelines (acceptable range: 0.8–1.25). This approach supports the use of TS as a reliable method for establishing equivalence among topical dermatological products not intended for systemic absorption. The research extensively examined products containing clotrimazole, clobetasol propionate, and later extended to acyclovir [4,5,11]. Through the rigorous application of this methodology, there is significant evidence supporting the use of TS as a valuable tool for the BE assessment of topical clotrimazole [5], clobetasol propionate [4], and acyclovir [11], and potentially other similar topical products when conducted with sufficient statistical power and appropriate normalization techniques [4,5,11]. This approach holds the promise of obviating the need for expensive and time-consuming clinical trials for such products. The incorporation of TEWL measurements for normalizing SC thickness is shown to be crucial in accounting for individual variations in skin barrier properties, thereby enhancing the method’s ability to accurately compare drug uptake. Furthermore, the research indicates a good correlation between the findings obtained from TS studies and the human skin blanching assay (HSBA) for topical corticosteroids [4], suggesting that TS could serve as a reliable surrogate measure for BA and BE.

In 2020, Rath and Kanfer [23,30] advanced a more efficient single time point approach, reasoning that for drugs acting within the SC, clearance data are not therapeutically warranted. Their method emphasizes direct measurement of drug uptake and incorporates several refinements: E_max_ modeling to optimize dose duration, TEWL-based normalization to account for differences in SC between subjects, and validation through self-reference and discriminatory power studies. They demonstrated that uptake-only TS is sufficient to establish BE for locally acting dermatological products, while reducing study complexity, subject, and producer burden. It is further supported by a standardized pilot–pivotal study design to identify an appropriate dose duration and incorporates defined procedures for randomization, TS, drug recovery, and statistical evaluation of the test/reference ratio using 90% confidence intervals [23].

### 3.2. Versatility in Skin Research

Another set of publications [31,32,33] has employed TS for a broader spectrum of applications, including the measurement of dermal exposure to various substances, the investigation of skin barrier function and disruption, and the evaluation of the removal of the SC itself, amongst others. A particular aspect of those papers involved developing and testing TS as a noninvasive sampling method for quantifying dermal exposure to substances like multifunctional acrylates as well as topically applied pharmaceutical agents [31]. Furthermore, TS was utilized to explore the influence of various parameters, such as pressure, application time, anatomical site, and the type of adhesive tape used, on acute barrier disruption and the cohesion of the SC [32]. Additionally, the publications described the extension of the TS method to investigate the potential of the method to mimic skin damage associated with grooming practices and to compare different evaporimetric methods for measuring transepidermal water loss on human skin that has undergone TS [32]. The methodology described varies and is based on the study objective, including the number of tape strips (ranging from 1 to 30), the type of adhesive tape, and the pressure application techniques. In addition to tape weighing, protein assays and spectroscopic measurements were used to quantify SC removal [33,34,35]. The flexibility of the approach highlights the broad applicability of TS beyond traditional BE studies. Furthermore, the investigations concerning dermal exposure often employed a limited number of consecutive tape strips, such as one or two, to capture the substance present on the surface and in the immediate subsurface layers of the skin [31]. In contrast, studies examining barrier disruption or mimicking shaving involved a larger number of strips, up to 20 or 30 [33]. Those also explore the effects of different methods for applying pressure (e.g., roller, stamp, thumb, stretched skin) and the total duration of the applied pressure (e.g., 2 s, 10 s) on the outcome of TS [33].

Comparison of the research objectives and associated methodologies described in Section 3.1 and Section 3.2 above reveals distinct primary focuses. The studies undertaken at BRI [4,5,11,23] are predominantly concerned with BE assessment of topical drug products for regulatory purposes, aiming to validate TS as a reliable in vivo method for comparing different formulations by quantifying drug penetration into the SC. In contrast, other research [31,32,33,34,35] encompasses a broader range of applications. Procedural variations between the two groups are also notable. The researchers at the BRI [4,5,11,23] typically employ a specific number of tape strips (around 15) to obtain a drug concentration profile across the SC for BE determination, whereas other studies [31,32,33,34,35] used a varied number of strips depending on the research question, using fewer strips (e.g., one or two) for initial dermal exposure assessments and more (up to 20 or 30) for examining barrier disruption or mimicking skin damage. While both groups utilize tape weighing, Guy et al. [31,32,33,34,35] placed a greater emphasis on quantifying the amount of SC removed using various methods such as tape weighing, protein assays, and spectroscopic techniques [33,34,35]. The primary focus of the researchers at the BRI [4,5,11,23] is on quantifying the drug content within the removed SC layers, using TEWL measurements for normalization rather than direct SC mass quantification. The data analysis approaches also differed, where in the former instance the focus is on PK parameters such as AUC and C_max_ derived from drug concentration profiles for statistical comparison of formulations in BE studies whereas the latter’s data analysis [31,32,33,34,35] varied based on the study’s objective, including calculating the percentage of deposited substance removed, assessing changes in TEWL and other skin physiology parameters, or correlating SC removal with protein content or spectroscopic measurements.

The implications of the different approaches by both groups are significant. The standardized method, incorporating TEWL normalization and detailed PK analysis, has important ramifications for the pharmaceutical industry and regulatory bodies in the context of topical drug product development and approval [4,5,11,23,36]. This approach lays the groundwork for utilizing TS as a potentially reliable and cost-effective alternative to extensive clinical trials for demonstrating BE. Conversely, the broader exploration [31,32,33,34,35] of TS’s applications has expanded its utility beyond regulatory science. Whereas the research on quantifying dermal exposure is relevant to occupational health and safety [31], the investigations into the effects of procedural parameters and the development of alternative quantification methods contribute to a fundamental understanding of skin physiology and the mechanics of TS [32,33,34,35].

### 3.3. Regulatory Recognition and Acceptance of TS

In 2003, the Japanese Ministry of Health, Labor and Welfare (MHLW) issued the “Guideline for BE Studies of Generic Products for Topical Use” [37], and the European Medicines Agency (EMA) Committee for Medicinal Products for Human Use (CHMP) adopted their September 2024 updated guideline that came into effect in April 2025 [10]. To date, neither the US FDA nor Health Canada has approved TS as an acceptable, generally applicable method for BE assessment of products for topical use. Ironically, in 1998, the US FDA issued a draft guidance outlining the TS methodology for the BE application of topical products [38]. However, in May 2002, the FDA withdrew the guidance as a result of concerns raised regarding the reproducibility of the method. These concerns were based on contradictory results generated by two reputable independent laboratories regarding the BE assessment of tretinoin gel products [39,40]. Hence, the DPK approach using tape stripping was largely discarded by the FDA as a viable option to pursue for the BE assessment of topical products. Various concerns/issues primarily based on the use of appropriate dose durations and variability in SC characteristics, especially the thickness of the SC, between individuals, were considered as major deficiencies, amongst others. Importantly, these deficiencies have subsequently been resolved by the efforts to establish the aforementioned reproducible single time point uptake procedure.

### 3.4. The Clearance Debate

The EMA guideline recommends a two time point design that incorporates both uptake and clearance phases [10]. Optimal uptake and clearance times are product-specific and should be established in pilot studies. Uptake should be sufficiently long to achieve diffusional steady state, as indicated by a plateau in drug mass recovered from the SC. Clearance should allow measurable drug loss from the SC, preferably ≥25% relative to uptake, and should not exceed 48 h to avoid desquamation effects. All sampling times should be carefully justified. In the uptake phase, sampling is performed immediately after product application and removal, capturing the total amount of drug absorbed into the SC during exposure. In the clearance phase, a second sampling is performed following a defined washout interval, typically between six and twenty-four hours later, to evaluate the amount of drug remaining in the SC and the extent of its elimination through metabolism, diffusion into deeper layers, or desquamation. Together, these two measurements allow the derivation of PK-like parameters such as an equivalent of C_max_ (maximal uptake in the SC), AUC (integrated uptake and clearance), and half-life or T_max_ (rate of clearance) [2]. From the regulatory perspective, clearance data may be necessary for drugs whose therapeutic targets lie deeper in the viable epidermis or dermis, where differences in elimination kinetics could influence local drug availability [2,41]. Such additional information is not necessary for drugs where the target site remains in close subcutaneous areas, such as the SC.

However, the EMA framework [10] also introduces significant limitations. Potential variability in uptake and clearance time selection, as well as limited harmonization of skin thickness across subjects, may contribute to overall study variability. The requirement for two separate sampling sessions per subject increases complexity, logistical burden, and subject discomfort. Moreover, clearance data can add variability, since desquamation rates and skin physiology differ both between and within subjects over time [2,41,42]. Thus, while the two time point method maximizes regulatory confidence by capturing both absorption and elimination, it does so at the expense of greater variability and unnecessary study burden.

### 3.5. Japanese DPK Guidance

According to the Japanese DPK guidance, drug recovery from the tapes is carefully standardized, typically involving validated extraction and analytical methods to ensure quantitative accuracy. The statistical evaluation mirrors systemic BE studies: the test/reference ratio of drug amounts in the SC is calculated, and 90% confidence intervals must fall within the conventional 80–125% acceptance range to demonstrate BE [37].

Overall, the Japanese guidance provides a highly standardized and pragmatic approach. By focusing on a single time point and emphasizing normalization strategies, it reduces study complexity and subject burden compared with dual time point designs, while still retaining the regulatory rigor of a pilot–pivotal structure and confidence interval-based equivalence testing. The procedures and approach outlined in the Japanese guidance are in accordance with the TS method developed by the researchers at BRI [4,5,11,23].

## 4. Comparative Methodological Analysis

Table 1 provides a comparison of the different types of TS methodologies. The evolution of TS methodologies for topical BE reflects a continuum of scientific innovation and regulatory perspectives, ranging from highly standardized PK analogs to versatile exploratory applications. The protocols developed at the BRI [4,5,11] focus on single time point uptake measurements, supported by pilot studies to optimize application time, controlled dosing on the volar forearm, and normalization by transepidermal water loss (TEWL) to account for variability in SC thickness. By calculating the area under the curve (AUC) of drug uptake across the SC and applying 90% confidence interval testing of the test/reference ratio, the research performed at the BRI [4,5,11] demonstrates that TS could reliably establish BE for products containing ketoprofen, clotrimazole, clobetasol propionate, acyclovir, and similar drugs targeting the SC [4,5,11].

Building on this foundation, Rath and colleagues from the same group advanced a more streamlined approach for SC-targeted drugs, arguing that clearance data add little value in such cases [23]. Their refinement incorporated E_max_ modeling to optimize dose duration, further emphasized TEWL normalization, and validated uptake-only designs through self-reference and discriminatory power studies [23]. This progression illustrates how rigorous validation can evolve into more efficient, context-specific methodologies that maintain sensitivity while reducing subject burden and study complexity.

Regulatory guidance provides another dimension. Whereas the EMA guideline [10] prescribes a dual time point design, such a conservative framework, which maximizes regulatory confidence, also introduces variability, subject burden, and complexity. By contrast, the Japanese DPK guidance [37] codifies a pragmatic single time point design at steady state or a fixed post-application interval, using either fixed or TEWL-guided strip numbers and normalization by SC weight or thickness. Its structured pilot–pivotal framework ensures reproducibility, with BE determined using the conventional 90% confidence interval approach.

Taken together, these perspectives illustrate the range of TS applications: On the one hand, some of the studies [31,32,34,35] highlight scientific versatility, whereas the single time point studies [4,5,11] established its regulatory credibility. Furthermore, Rath et al. [23] refined it for SC-targeted efficiency, while the EMA [10] and Japan [37] represent divergent regulatory philosophies: one conservative and one pragmatic. The cumulative evidence supports a fit-for-purpose paradigm: for drugs acting primarily in the SC, uptake-only designs are scientifically justified and operationally efficient; for drugs requiring deeper penetration, additional kinetic characterization, including clearance, may still be warranted to ensure therapeutic equivalence.

Several researchers pursued a broader vision of TS beyond regulatory BE, applying it to investigate dermal exposure, skin barrier function, and grooming-related skin damage. These studies adopted flexible protocols varying in strip number, adhesive type, pressure, and quantification methods (e.g., protein assays, spectroscopy) [31,32,34,35]. Although not designed for regulatory submissions, their work highlights the versatility of TS as a research tool in toxicology, occupational health, and skin physiology.

## 5. Critical Evaluation of Clearance Time Measurements

### 5.1. Theoretical Justification

Clearance-phase TS is designed to evaluate the elimination of drug from the SC over time, providing insights into drug retention, degradation, or further penetration. This may be particularly relevant for drugs intended to act in deeper skin layers and more relevant for transdermal delivery systems. Pharmacokinetic modeling in such cases benefits from both uptake and clearance data to understand temporal drug dynamics.

### 5.2. When Clearance Is Redundant

For topical drugs whose therapeutic action is localized within the SC or upper epidermis, clearance-phase measurements do not contribute to understanding or confirming clinical efficacy. In these cases, drug elimination is often governed by natural skin turnover, such as desquamation, rather than pharmacological clearance. Examples include antifungals such as clotrimazole or keratolytic agents such as salicylic acid, where drug retention in the SC is desired and adequate for therapeutic action. Measuring only the initial uptake is therefore sufficient to establish BE for such drugs, simplifying the study design while maintaining relevance.

**Figure 2:**
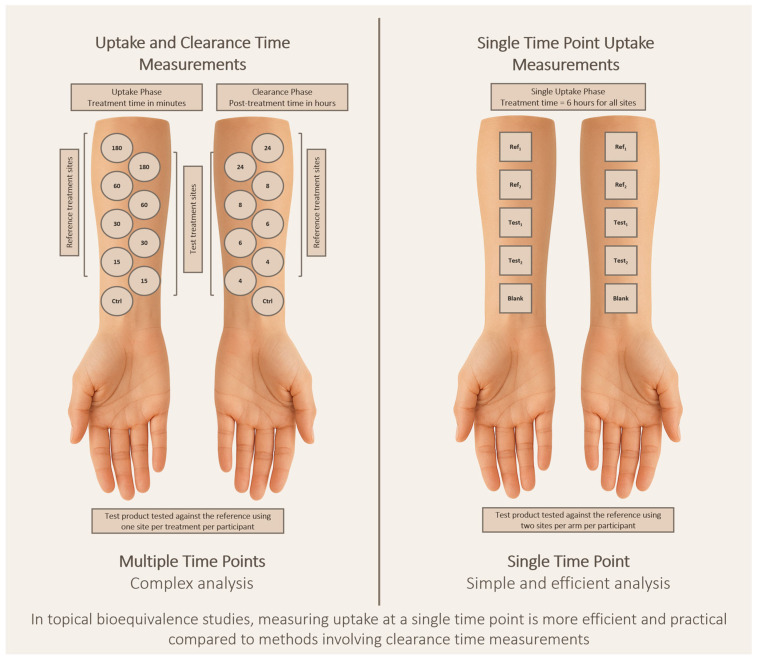
Comparison of multiple time points and single time point product application methods.

**Figure 3:**
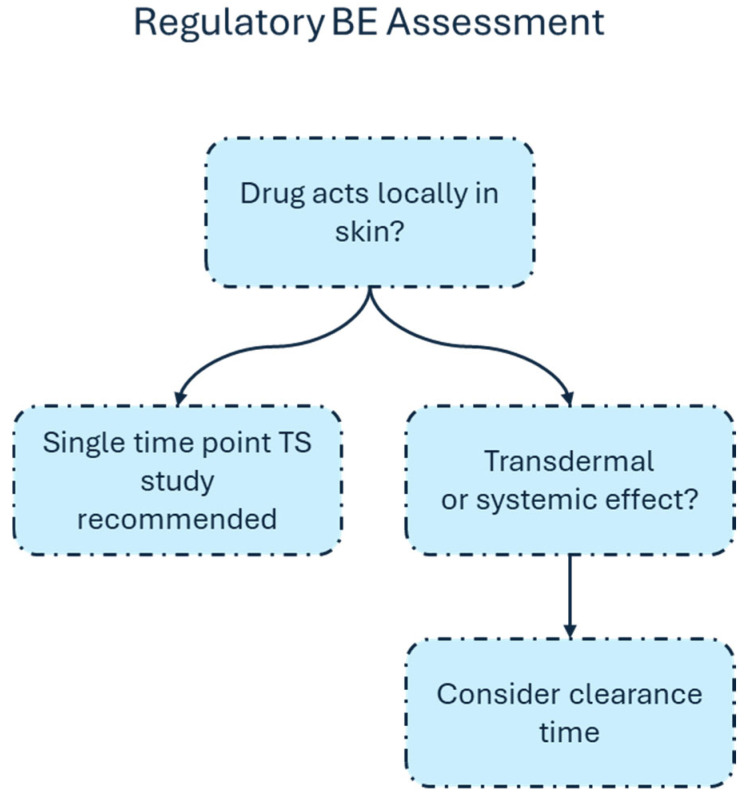
Framework for selecting an appropriate study design for regulatory BE assessment of topical products using TS.

Figure 2 provides a conceptual comparison of tape stripping study designs for assessing topical BE. The illustration compares single time point uptake-only tape stripping methodologies with two time point uptake-clearance designs. The figure highlights key procedural elements, including dose application and SC sampling. For topical therapies used in the treatment of dermatological conditions such as psoriasis, atopic dermatitis, acne, and superficial fungal infections that act primarily within or near the SC meaningful [4,5,6,7,8,9], uptake-only designs capture the relevant exposure at the site of action and provide a scientifically justified and operationally efficient approach for regulatory BE assessment. Clearance measurements, which increase study complexity and variability, may be warranted only when deeper epidermal or dermal targets are relevant, such as with diclofenac and similar anti-inflammatory drugs which are used when site of action is regional and not local [43]. Therefore, we propose a decision tree depicted in Figure 3 to support the selection of study design for regulatory BE assessment using TS, distinguishing when a single time point study is sufficient versus when multiple time points with clearance assessment are necessary.

## 6. “Model-Based” Innovation and Artificial Intelligence (AI) Insights

Ozdin et al. [44] introduced a DPK method that simplifies TS for assessing the BE of topical products while enabling direct estimation of PK parameters. The approach uses a single discriminative dose duration determined with an E_max_ model, followed by sequential TS in which each layer serves as a surrogate time point to generate a depth–time profile across the SC. These data are analyzed with population PK modeling (ADAPT5^®^, MLEM algorithm) to derive two key parameters: K_in_, representing the rate of drug input into the skin, and F_S_, quantifying the extent of absorption. Together, these systemic PK–like metrics provide a robust characterization of topical drug delivery, offering greater interpretability than descriptive endpoints such as the Relative Depth method or uptake-only AUC analyses. The framework successfully distinguished between bioequivalent and bioinequivalent formulations, demonstrating superiority compared with the FDA’s earlier withdrawn two-time point design [38] while also reducing sampling demands, eliminating the need for clearance phases, and requiring fewer subjects. By integrating TS with population PK modeling, the method delivers richer, interpretable PK-like measures and provides a regulatory-aligned, efficient alternative to conventional multiple time point or assumption-heavy approaches, thereby reducing study complexity and strengthening confidence in BE decisions.

Integration of TS data with artificial intelligence (AI) and machine learning (ML) models holds promise for personalized DPK profiling. It presents a transformative opportunity where AI algorithms could probably be trained on large datasets to predict dermal permeation and drug transit and support barrier integrity and BE conclusions. By using “TEWL-based normalization” to account for individual variations in skin barrier properties and SC thickness, AI could theoretically analyze these datasets to provide personalized insights into how a specific patient’s skin physiology affects drug penetration.

## 7. Conclusions

Tape stripping is a versatile and minimally invasive method for assessing topical drug BE. The TS methods employed by the researchers at BRI [4,5,11,23], Japanese Guideline [37], EMA Guideline [10], and others [31,32,34,35], while relying on the same fundamental technique, are tailored to address distinct research objectives. The research work carried out at BRI [4,5,11,23] is instrumental in exploring the potential of TS for regulatory BE assessment through a meticulously standardized approach that focuses on drug quantification and PK analysis. The EMA [10] and other similar approaches [31,32,34,35], on the other hand, have broadened the scope of TS’s application, demonstrating its versatility in quantifying dermal exposure, investigating skin barrier properties, and even mimicking physiological processes, but the approach to include the assessment of clearance to declare BE is questionable. The inclusion of clearance measurements should be reserved for scenarios where they meaningfully contribute to understanding drug disposition. For the majority of locally acting topical agents, uptake-only TS should be adequate, efficient, and scientifically justified for regulatory BE assessments. In light of the foregoing, an important objective is standardization and subsequent regulatory acceptance. Hence, a primary future direction is resolving “methodological disparities” to gain broader regulatory approval from agencies like the US FDA and Health Canada, which currently do not accept TS as a generally applicable method.

## Figures and Tables

**Table 1:** Comparative table of TS methodologies.

Method	Versatility in Skin Research [2,14,15,29,31,33,34,35]	BRI Protocols [4,5,11]	BRI Refinement [23]	EMA Guideline [10]	Japanese DPK Guidance [37]
Design	Highly flexible; study-specific	Single time point uptake with pilot + pivotal studies	Uptake-only refinement for SC-targeted drugs	Two time point (uptake + clearance); application times determined through pilot studies and justified case-by-case basis	Single time point (steady state or fixed interval)
Primary Focus	Dermal exposure, barrier function, and grooming-related damage	Establish a regulatory grade BE methodology	Streamline BE for SC-targeted drugs	Regulatory conservatism; mimics systemic PK	Regulatory pragmatism; efficient BE
Tape Stripping	1–30 strips; varied adhesives, pressure, anatomical sites	~15–20 strips; volar forearm; standardized protocol	~15–20 strips; volar forearm; standardized protocol, fixed or TEWL-guided strips; uptake only	Fixed strip number; TEWL normalization recommended	Fixed or TEWL-guided strips
Normalization	SC removal quantified (tape weighing, protein assays, spectroscopy)	SC removal quantified, TEWL correction for SC thickness	SC removal quantified, TEWL correction for SC thickness emphasized; E_max_ modeling for dose duration	TEWL or strip number for SC depth	SC weight or thickness; TEWL-guided
Study Framework	Flexible, exploratory; not BE-driven	Pilot to optimize dose duration (E_max_ modeling); pivotal with 90% CI BE testing	Pilot to optimize dose duration (E_max_ modeling); pivotal with 90% CI BE testing, uptake-only validated with self-reference and discriminatory power	Crossover; standardized dosing; statistical BE with 90% CI	Pilot–pivotal structure; standardized randomization, stripping, recovery
Endpoints	Exposure levels, barrier disruption, TEWL, SC cohesion	AUC of drug uptake (TEWL-corrected and uncorrected)	AUC of drug uptake (TEWL-corrected and uncorrected)	C_max_, AUC, half-life equivalents from uptake + clearance	Uptake AUC at single time point; 90% CI
Advantages	Broad applicability; informs toxicology, occupational health, and skin physiology	Rigorous, reproducible, regulatory credibility; tested on multiple drugs	Rigorous, reproducible, regulatory credibility; efficient for SC-targeted drugs; reduced complexity and burden	Conservative, comprehensive; regulatory confidence	Standardized, pragmatic; reduced burden and variability
Limitations	Not standardized; less relevant for regulatory BE	More strips, optimization required, may face skepticism for deeper-acting drugs	May face skepticism for deeper-acting drugs	Potential variability in uptake and clearance time selection; limited harmonization of skin thickness across subjects; increased study complexity; differing views on the relevance of clearance measurements	May miss clearance kinetics for deeper targets

## Data Availability

No new data were created or analyzed in this study.

## Abbreviations

The following abbreviations are used in this manuscript:

AUC	Area Under the Curve
BA	Bioavailability
BE	Bioequivalence
BRI	Biopharmaceutics Research Institute
CHMP	Committee for Medicinal Products for Human Use
C_max_	Maximum Concentration
DPK	Dermatopharmacokinetics
EMA	European Medicines Agency
E_max_	Maximum Effect Model
FDA	United States Food and Drug Administration
F_S_	Fraction Absorbed into Skin
HSBA	Human Skin Blanching Assay
HPLC	High Performance Liquid Chromatography
K_in_	Rate of Drug Input into the Skin
MHLW	Ministry of Health, Labour and Welfare (Japan)
MLEM	Maximum Likelihood Expectation Maximization
PK	Pharmacokinetics
SC	Stratum Corneum
TEWL	Transepidermal Water Loss
TS	Tape Stripping
